# Dithiocarbamates as Effective Reversible Addition–Fragmentation Chain Transfer Agents for Controlled Radical Polymerization of 1-Vinyl-1,2,4-triazole

**DOI:** 10.3390/polym14102029

**Published:** 2022-05-16

**Authors:** Alexander S. Pozdnyakov, Nadezhda P. Kuznetsova, Tatyana A. Semenova, Yuliya I. Bolgova, Anastasia A. Ivanova, Olga M. Trofimova, Artem I. Emel’yanov

**Affiliations:** A.E. Favorsky Irkutsk Institute of Chemistry, Siberian Branch of the Russian Academy of Sciences, 1 Favorsky Str., Irkutsk 664033, Russia; nkuznetsova@irioch.irk.ru (N.P.K.); semjenova@irioch.irk.ru (T.A.S.); omtrof1@irioch.irk.ru (Y.I.B.); ivanova93@irioch.irk.ru (A.A.I.); omtrof@irioch.irk.ru (O.M.T.); emelyanov@irioch.irk.ru (A.I.E.)

**Keywords:** controlled radical polymerization, 1-vinyl-1,2,4-triazole, chain transfer agents, dithiocarbamates, xanthates, trithiocarbonates

## Abstract

Narrow dispersed poly(1-vinyl-1,2,4-triazole) (PVT) was synthesized by reversible addition–fragmentation chain transfer (RAFT) polymerization of 1-vinyl-1,2,4-triazole (VT). AIBN as the initiator and dithiocarbamates, xanthates, and trithiocarbonates as the chain transfer agents (CTA) were used. Dithiocarbamates proved to be the most efficient in VT polymerization. Gel permeation chromatography was used to determine the molecular weight distribution and polydispersity of the synthesized polymers. The presence of the CTA stabilizing and leaving groups in the PVT was confirmed by ^1^H and ^13^C NMR spectroscopy. The linear dependence of the degree of polymerization on time confirms the conduct of radical polymerization in a controlled mode. The VT conversion was over 98% and the PVT number average molecular weight ranged from 11 to 61 kDa. The polydispersity of the synthesized polymers reached 1.16. The occurrence of the controlled radical polymerization was confirmed by monitoring the degree of polymerization over time.

## 1. Introduction

The development of modern innovative biomedical technologies is closely related to the creation of the latest functional polymeric materials with a complex of valuable and practically significant properties. The most important characteristics of materials are their pharmacological activity, hydrophilicity, non-toxicity, biocompatibility, resistance to aggressive media, and heat resistance. Polymers of 1-vinyl-1,2,4-triazole possess these properties to the full extent. Poly(1-vinyl-1,2,4-triazole) (PVT) is a unique biocompatible water-soluble non-toxic polymer (LD_50_ > 5000 mg/kg) [1,2,3] that is chemically and thermally stable [4]. PVT has a complexing [5], stabilizing, and flocculating ability [6]. Recently, acid-doped PVTs have been studied as promising proton-conducting polymer electrolytes in proton-exchange membrane fuel cells [7,8], dielectric layer for organic field effect transistors [9]. Poly(1-vinyl-1,2,4-triazole) is promising for the creation of modern biologically active materials used for the biosynthetic activation of connective tissue cells of the body [10] as a hydrophilic material with a low refractive index for soft contact lenses [11]. PVT has shown high efficiency as a stabilizing matrix for silver and gold nanoparticles [12,13]. On the basis of interpolymer complexes of PVT and polyacrylic acid, under the action of X-rays, nanocomposites with a controlled size of gold and copper nanoparticles were obtained [14,15]. Using the radiation–chemical approach, nanocomposites with silver nanoparticles in a PVT matrix were obtained that exhibited a high antimicrobial activity against various strains of bacteria (*E. coli*, *P. aeruginosa*, *K. pneumoniae*), which depends on the size of the nanoparticles [16].

An important characteristic of polymers for medical purposes is the molecular weight and polydispersity, which should be in the narrowest possible range. The controlled synthesis of narrowly dispersed polymers with a given structure and with a given molecular weight is a priority in modern polymer chemistry. In this regard, the development of methods for the effective control of the molecular weight of polymers based on 1-vinyl-1,2,4-triazole is a significant task. The most promising way to achieve these results is the use of reversible addition-fragmentation chain transfer (RAFT) polymerization and the control of the parameters of the molecular weight distribution in polymers [17,18,19]. Intensive studies in recent years have made it possible to take a fresh look at the synthetic possibilities of RAFT polymerization for the creation of functional macromolecules of various architectures and to expand our understanding of the mechanism of this process [18,19,20,21,22,23,24].

In controlled radical polymerization, along with the elementary chain initiation and propagation reactions, reversible chain transfer reactions also occur, which provide a uniform increase in the degree of polymerization of the active macromolecular chains. Such reversible addition–elimination reactions lead to a low degree of polydispersity of the obtained polymers and allow one to control their molecular weight [21]. It is known that chain transfer agents are used for the synthesis of narrowly dispersed polymers of various non-conjugated N- and O-vinyl monomers including the preparation of block copolymers [25,26,27,28,29,30,31]. Thus far, only one work is known on the use of VT in RAFT polymerization using a xanthate chain transfer agent to obtain amphiphilic block copolymers containing triazole and carbazole units [25].

The purpose of this work was to develop methods for the synthesis of narrowly dispersed poly-1-vinyl-1,2,4-triazole through controlled radical polymerization with reversible chain transfer by the addition–fragmentation mechanism in the presence of various chain transfer agents; study of the effect of the reaction conditions, initiator, and chain transfer agent concentrations on the molecular weight characteristics of poly-1-vinyl-1,2,4-triazole.

## 2. Materials and Methods

### 2.1. Materials

1-Vinyl-1,2,4-triazole (VT) was synthesized and purified according to the procedure [13]. Azobisisobutyronitrile (AIBN, 99%), cyanomethyl methyl(phenyl)dithiocarbamate (CTA1, 98%) were purchased from Sigma-Aldrich (Munich, Germany) and used as received without further purification. Cyanomethyl methyl(pyridine-4-yl)dithiocarbamate (CTA2), ethyl 2-[(ethoxycarbonothiolyl)thio]propanoate (CTA3), O-ethyl S-(1-phenylethyl)dithiocarbonate (CTA4), dibenzyl trithiocarbonate (CTA5), and 4-cyano-4-{[(ethylthio)carbonothioyl]thio})pentanoic acid (CTA5) were synthesized and purified according to the procedure [32,33,34,35,36]. Dimethylformamide (DMF), acetone, and methanol were distilled and purified according to the known procedures.

### 2.2. Procedure for the Synthesis of Chain Transfer Agent (CTA)

#### 2.2.1. Synthesis of Cyanomethyl Methyl(Pyridine-4-yl)Dithiocarbamate (CTA2)

CTA2 was synthesized according to the method in [32]. n-Butyl lithium (2.5 M, 22 mL, 55.00 mmol) was added to a solution of 4-(methylamino)pyridine (4.5 g, 42.05 mmol) in THF (100 mL) at −10 °C. Then, the reaction mixture was stirred for 1 h. Carbon disulfide (5.1 mL, 6.468 g, 84.00 mmol) was added at 0 °C, and the mixture was left to stir for 20 h at room temperature. Bromoacetonitrile (7.57 g, 63.00 mmol) was added to the resultant mixture at 0 °C and stirred at room temperature for 2 h. The reaction mixture was extracted with diethyl ether and washed with saturated sodium hydrogen carbonate and brine. The organic layer was dried over sodium sulfate. Then, the solvent was removed under reduced pressure. The CTA2 was purified by column chromatography using silica gel as the stationary phase and ethyl acetate–hexane (30:70) as the eluent. ^1^H NMR (CDCl_3_, δ, ppm): 8.73–8.68 (m, 2H, PyH-2,6), 7.21–7.17 (m, 2H, PyH-3,5), 4.06 (s, 2H, SCH_2_CN), 3.81 (s, 3H, NCH_3_); ^13^C NMR (CDCl_3_, δ, ppm): 194.00 (C=S), 149.35 (PyC-2,6), 143.58 (PyC-4), 123.37 (PyC-3,5), 116.06 (CN), 46.71 (NCH_3_), 23.34 (SCH_2_CN).

#### 2.2.2. Synthesis of Ethyl 2-[(Ethoxycarbonothiolyl)thio]propanoate (CTA3)

CTA3 was synthesized according to the method in [33]. Ethyl 2-bromopropionate (1.023 g, 5.7 mmol) was dissolved in 9 mL of ethanol. The solution was cooled to 0 °C and the potassium salt of O-ethyl xanthic acid (1.0 g, 6.2 mmol) was added. The reaction mixture was stirred for 24 h. The product was then extracted with ether/pentane (2:1), washed with distilled water, and dried over magnesium sulfate. The solvent was removed under reduced pressure. ^1^H NMR (CDCl_3_, δ, ppm): 4.60 (q, 2H), 4.33 (q, 1H), 4.16 (q, 2H), 1.53 (d, 3H), 1.38 (t, 3H), 1.25 (t, 3H); ^13^C NMR (CDCl_3_, δ, ppm): 212.10 (C=S), 171.32, 70.7, 61.7 (C=O), 47.19, 16.91, 14.11, 13.67 (SCH).

#### 2.2.3. Synthesis of O-Ethyl-S-(1-phenylethyl)dithiocarbonate (CTA4)

CTA4 was synthesized according to the method in [34]. The potassium salt of ethylxanthogenic acid (1.0 g, 6.2 mmol) was dissolved in 10 mL of ethanol at 57 °C. Then, 1-bromoethylbenzene (1.2 g, 6.2 mmol) was added dropwise. The reaction mixture was stirred for 5 h. Then, the reaction mixture was cooled to room temperature, and 30 mL of distilled water were added. The product was extracted with dry diethyl ether (3 × 25 mL). The organic layer was dried over MgSO_4_. The solvent was removed under reduced pressure. ^1^H NMR (CDCl_3_, δ, ppm): 7.41–7.28 (m, 5H, ArH), 4.92 (q, 1H), 4.63 (q, 2H), 1.73 (d, 3H, CH_3_CHS), 1.40 (tr, 3H, CH_3_CH_2_O); ^13^C NMR (CDCl_3_, δ, ppm): 213.4 (C=S), 141.84, 128.6, 127.5, 126.2, 69.4 (C_6_H_5_), 49.3, 21.8, 13.8 (SCH).

#### 2.2.4. Synthesis of Dibenzyl Trithiocarbonate (CTA5)

CTA5 was synthesized according to the method in [35]. Carbon disulfide (1.0 g, 13.13 mmol) was added to a solution of benzyl chloride (1.6 g, 12.5 mmol) in DMF (12.5 mL). Potassium carbonate (1.7 g, 12.5 mmol) was added to the resulting mixture at 25 °C. The mixture was stirred at 40 °C for 24 h. Then, the reaction was stopped by pouring the mixture into ice water. The product was extracted with ethyl acetate, and dried over anhydrous sodium sulfate. The solution was then decanted and the solvent was removed under reduced pressure. The dibenzyl trithiocarbonate was purified by silica gel column chromatography using hexane as the eluent. ^1^H NMR (CDCl_3_, δ, ppm): 7.37–7.29 (10 H, ArH), 4.67 (s, 4 H, SCH_2_); ^13^C NMR (CDCl_3_, δ, ppm): 222.55 (C=S), 134.86, 129.19, 128.63, 127.71 (C_6_H_5_), 41.5 (SCH_2_).

#### 2.2.5. Synthesis of 4-Cyano-4-{[(ethylthio)carbonothioyl]thio}pentanoic Acid (CTA6)

CTA6 was synthesized according to the method in [36]. Ethanthiol (2.36 g, 38 mmol) was added to a suspension of sodium hydride (60% in oil) (1.58 g, 39.5 mmol) in diethyl ether (90 mL) with stirring from 0 to 5 °C. Then, carbon disulfide (3.01 g, 39.5 mmol) was added to the reaction mixture at 0 °C. The sodium S-ethyl trithiocarbonate that formed was filtrated and used in the next step. To a suspension of sodium S-ethyltrithiocarbonate (3.93 g, 24.5 mmol) in diethyl ether (70 mL) was added iodine (3.15 g, 12.5 mmol). The reaction mixture was stirred at room temperature for 1 h. The formed precipitate was removed by filtration. The filtrate was washed with aqueous sodium thiosulfate and dried over sodium sulfate. The bis(ethylsulfanylthiocarbonyl) disulfide was then isolated by rotary evaporation. A solution of 4,4′-azobis(4-cyanopentanoic acid) (1.05 g, 37.5 mmol) and bis(ethylsulfanylthiocarbonyl) disulfide (0.69 g, 2.5 mmol) in ethyl acetate (30 mL) was heated at reflux for 18 h. Next, the solvent was removed under reduced pressure. The CTA6 was isolated by column chromatography using silica gel as the stationary phase and ethyl acetate–hexane (50:50) as the eluent. ^1^H NMR (CDCl_3_, δ, ppm): 3.31 (q, 2H, SCH_2_CH_3_), 2.63–2.35 (m, 4H, CH_2_CH_2_), 1.85 (s, 3H, CCH_3_), 1.33 (t, 3H, SCH_2_CH_3_); ^13^C NMR (CDCl_3_, δ, ppm): 216.55 (C=S), 177.37 (C=O), 118.65 (C≡N), 46.10 (SCCN), 33.35 (CCH_2_), 31.31 (SCH_2_CH_3_), 29.47 (CH_2_C(O)OH), 24.71 (CCH_3_), 12.65 (SCH_2_CH_3_).

#### 2.2.6. Free Radical Polymerization of 1-Vinyl-1,2,4-Triazole

PVT was synthesized by the free radical polymerization of VT in DMF in degassed ampoules in the presence of the initiator azobis(isobutyronitrile) (1.2 mass%). 1-Vinyl-1,2,4-triazole (0.1 g, 1.05 mmol), DMF (0.2 mL), and AIBN (0.0012 g, 0.0073 mmol) were placed in a tube. The mixture was stirred and kept in a thermostat at 60 °C for 24 h until the completion of polymerization. The resulting transparent block was dissolved in DMF, precipitated twice with an ethanol–acetone mixture (1:2), and dried in a vacuum over P_2_O_5_ at 50 °C to constant weight. PVT was obtained with a yield of 76%, M_n_ 157 kDa, and PDI 2.02. FT-IR (ν, cm^–1^): triazole—1506 (C=N), 1435 (C–N), 1277 (N–N), 1004 (C–H), 660 (C–N); polymer chain—3111 (C–H), 2973 (CH_2_). ^1^H NMR (D_2_O, δ, ppm): 8.09–7.50 (br m, 2H, triazole ring), 4.11–2.70 (br m, 1H, CH in the polymer main chain), 2.30–1.75 (br, 2H, CH_2_ in the polymer main chain); ^13^C NMR (D_2_O, δ, ppm): 152.6–150.9, 143.4–141.9 (CH, triazole ring), 57.0–53.7 (CH in the polymer main chain), 37.7–35.3 (CH_2_ in the polymer main chain); ^15^N NMR (D_2_O, δ, ppm): −159.4 (N-1), −136.4 (N-4), −96.5 (N-2).

#### 2.2.7. Controlled Radical Polymerization of 1-Vinyl-1,2,4-Triazole

PVT was synthesized by the controlled radical polymerization of VT in methanol or DMF in degassed ampoules in the presence of AIBN as the initiator and chain transfer agent CTA at 60 °C for 24 h. The yield of the resulting polymer was equal to 86%.

The general procedure (for example, CTA2) was as follows: VT (0.1 g, 1.05 mmol), AIBN (0.00035 g, 0.0021 mmol), CTA2 (0.0023 g, 0.0105 mmol), and methanol (or DMF) were placed in a dry glass ampoule equipped with a magnetic stirrer. After 3-fold degassing by repeating freeze–thaw cycles in vacuum (residual pressure ~5 × 10^–5^ mm Hg), the ampoule was sealed. Then, the ampoule was placed in an air oven, and the reaction mixture was stirred at a temperature of 60 °C for 24 h. Next, the reaction was stopped by cooling the ampoule in liquid nitrogen. DMF (0.5 mL) was added to the resulting yellow viscous solution, stirred for 30 min, and precipitated with vigorous stirring into a cooled excess of acetone (20 mL). The formed precipitate was allowed to stand for three hours and separated by centrifugation at 7000 rpm. A fresh portion of chilled acetone was added twice to the precipitate and centrifuged again. Then, the precipitate was dried in a vacuum oven at room temperature to a constant weight for 24 h. PVT in the form of a fine white powder was obtained with a yield of 0.076 g (76%).

To monitor the polymerization reaction over time, a solution of VT, AIBN, CTA2, and DMF, which was dosed into six glass ampoules, was used. Each solution was degassed with three freeze–thaw cycles under vacuum. The ampoules were sealed under vacuum, placed in an air oven, and kept at 60 °C for 0.5–24 h with constant stirring. After reaching the specified polymerization time, the reaction was stopped by cooling the ampoule with liquid nitrogen. The conversion, number average molecular weight, and polydispersity of the homopolymer in the reaction mixture were determined by GPC.

To confirm the activity of polyCTA, further homopolymerization was studied in order to increase the degree of elongation of the macromolecular chain. For example, polyCTA, VT, and AIBN in dry DMF was placed in a dry ampoule and degassed by three freeze–thaw cycles in vacuum. Polymerization was carried out at 60 °C for 6 h. The reaction mixture was precipitated into an excess of chilled acetone, a finely dispersed powder was isolated, and then dried in a vacuum oven at room temperature to constant weight for 24 h.

### 2.3. Characterization

The FTIR spectra were recorded on a Varian 3100 FTIR spectrometer (Palo Alto, CA, USA) in the range of 400–4000 cm^−1^. ^1^H, ^13^C, and ^15^N NMR spectra were measured on a Bruker DPX-400 (Bruker, Bremen, Germany) and Bruker AV-400 (Bruker, Karlsruhe, Germany) at 400.13, 100.62, and 40.55 MHz, respectively, in CDCl_3_ and D_2_O. Chemical shifts are given relative to TMS (^1^H, ^13^C) and MeNO_2_ (^15^N). The 2D ^1^H–^15^N NMR spectra were recorded using the HMBC-gp ^1^H–^15^N correlation technique. The molecular weight of the polymer was determined by gel permeation chromatography using a Shimadzu LC-20 Prominence system (Shimadzu Corporation, Kyoto, Japan) fitted with a differential refractive index detector Shimadzu RID-20A and column Agilent PolyPore 7.5 mm × 300 mm (PL1113-6500) at 50 °C. N,N-Dimethylformamide solution was used as the eluent at the flow rate of 1 mL/min. Dissolution of the samples was performed at 50 °C for 24 h with stirring. The calibration was carried out using a series of polystyrene standards, Polystyrene High EasiVials (PL2010-0201), consisting of 12 samples with molecular weights from 162 to 6,570,000 g/mol.

## 3. Results and Discussion

### 3.1. Free-Radical Polymerization of 1-Vinyl-1,2,4-Triazole

Previously, it was reported that 1-vinyl-1,2,4-triazole easily polymerizes under free-radical polymerization conditions [37,38,39] in the presence of the AIBN as an initiator at 60 °C in DMF, DMAA, or water, in the monomer bulk. The use of redox systems (ammonium persulfate–urea) makes it possible to lower the reaction temperature to room temperature [37]. Radical polymerization of VT leads to the production of homopolymers with a yield of up to 96% in a wide range of molecular weights at the initiator concentrations of 1 × 10^−3^–3 × 10^−3^ mol/L.

**Scheme 1:**
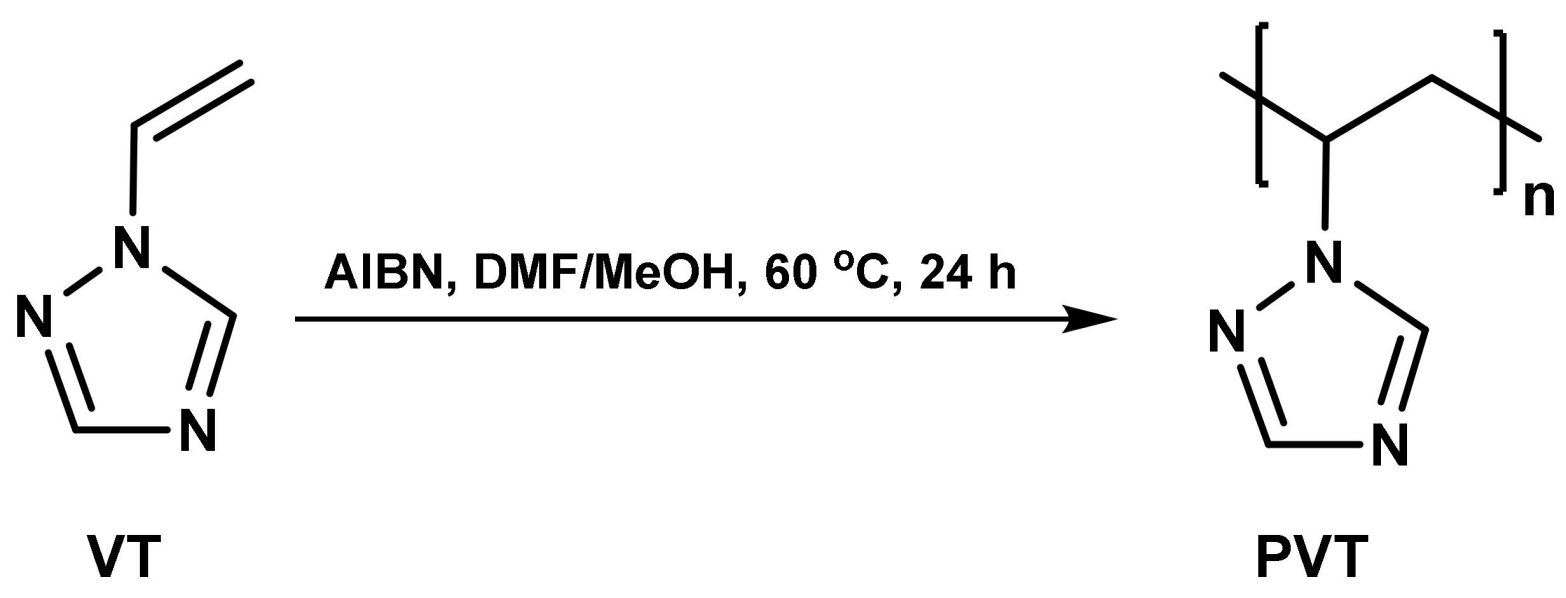
The synthesis of poly(1-vinyl-1,2,4-triazole) by free radical polymerization.

To compare the molecular weight characteristics of the homopolymer obtained by free-radical and controlled radical polymerization, PVT was synthesized without the use of chain transfer agents (Scheme 1).

As a result of VT polymerization in the presence of the AIBN initiator, a high-molecular-weight homopolymer with M_n_ 136 kDa in MeOH and 176 kDa in DMF was formed, the conversion being 92 and 87%, respectively. The resulting polymers were characterized by a polydispersity of 2.19 in MeOH and 2.10 in DMF, which indicates a significant scatter of PVT macromolecules in the degree of polymerization. The homopolymer dissolved well in polar solvents DMF, DMSO, methanol, and water and was insoluble in acetone, hexane, ethyl alcohol, and chloroform.

### 3.2. Controlled Radical Polymerization of 1-Vinyl-1,2,4-Triazole

**Scheme 2:**
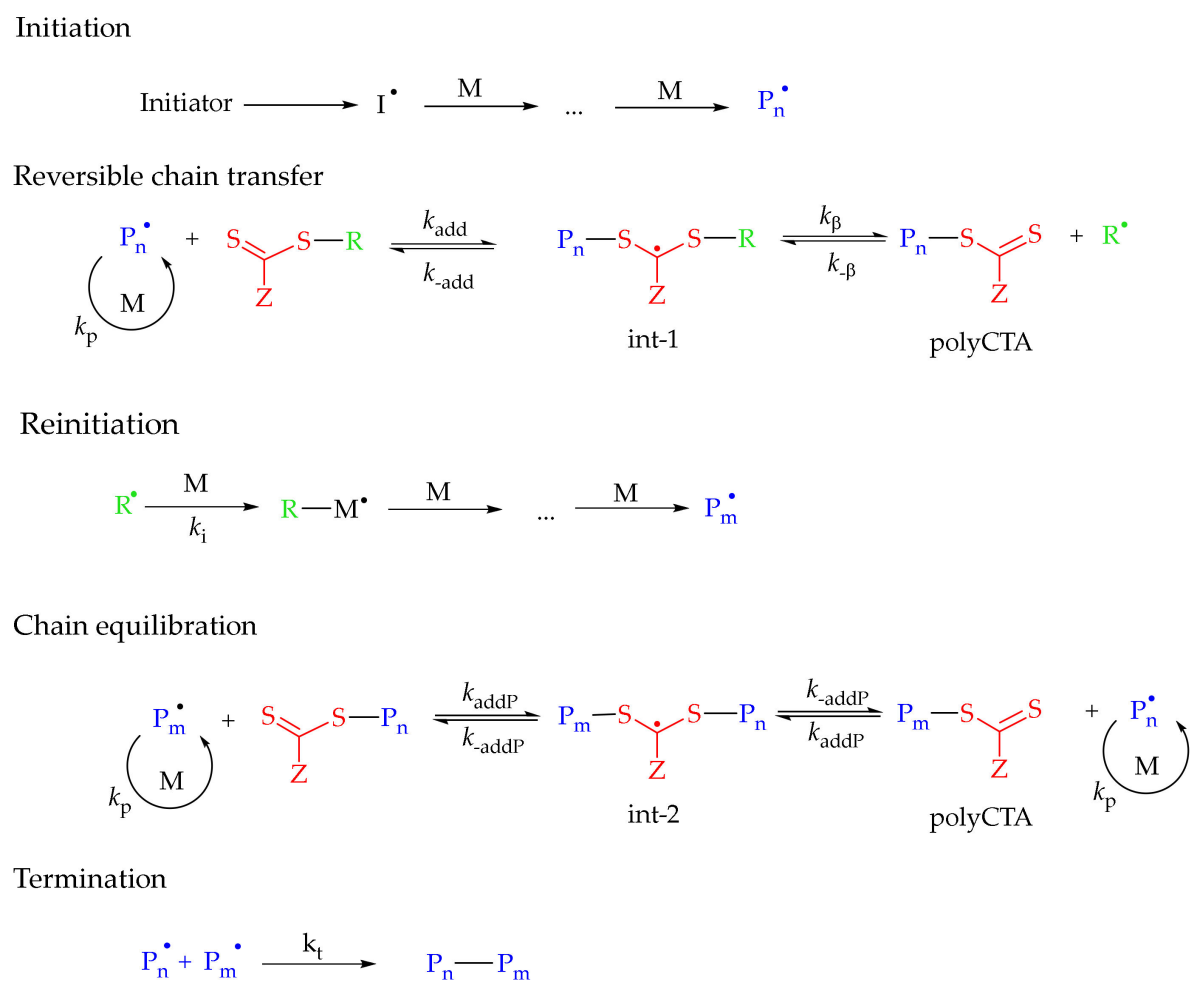
Mechanism of the controlled radical polymerization.

It is known that the controlled radical polymerization of N- and O-vinyl monomers is a difficult task, since the resulting monomeric radicals are highly reactive due to their non-conjugated kind and the strong electron-donating properties of the functional groups [40,41,42]. Controlled radical polymerization uses various chain transfer agents (CTAs) such as dithiocarbamates, dithioethers, xanthates, and trithiocarbonates. CTAs provide the control of polymerization through reversible chain transfer reactions, minimizing the instantaneous concentration of radicals and reducing the probability of chain termination reactions [43] (Scheme 2).

**Figure 1:**
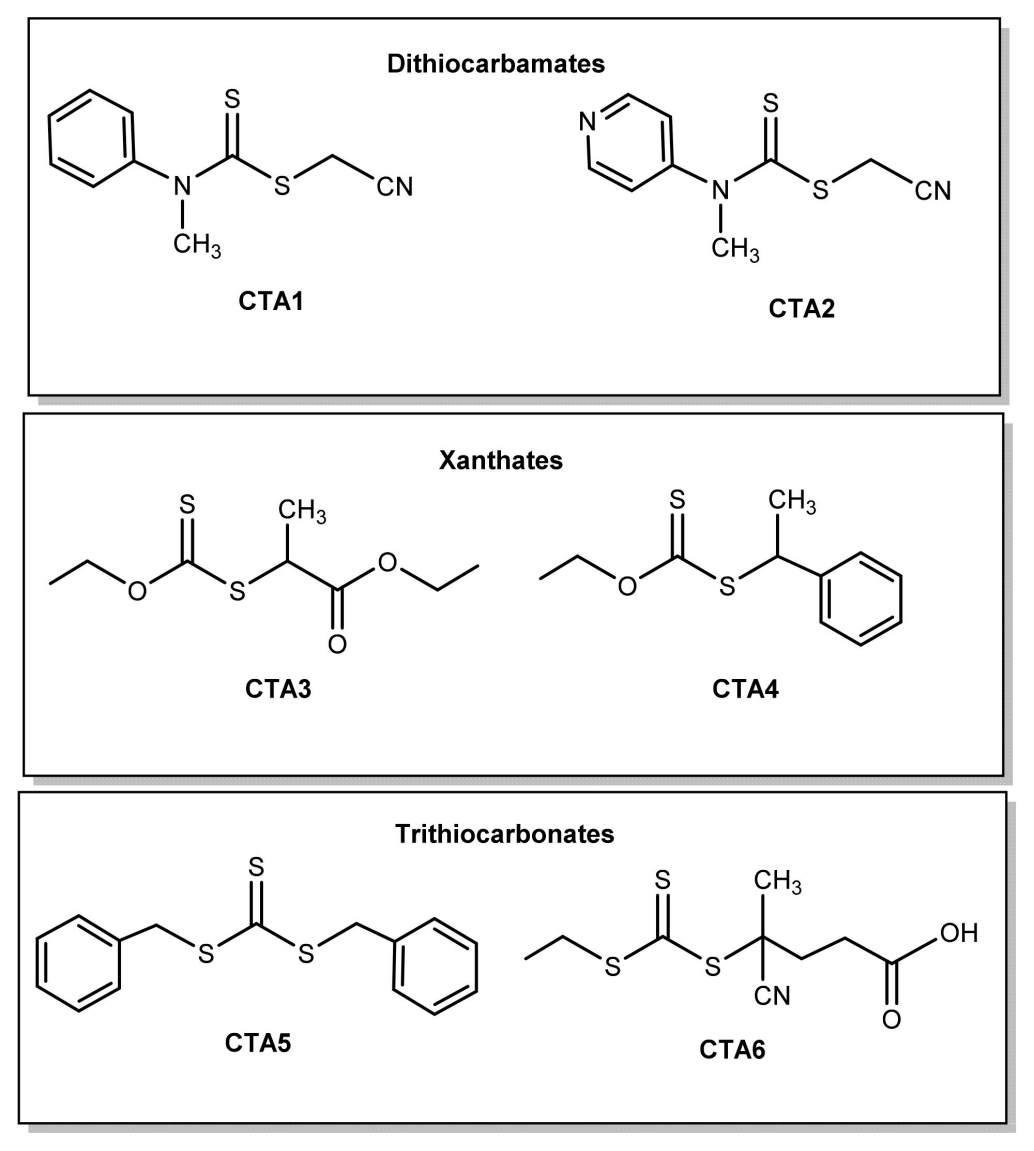
Chain transfer agent: CTA1—cyanomethyl methyl(phenyl)dithiocarbamate; CTA2—cyanomethyl methyl(pyridine-4-yl)dithiocarbamate; CTA3—ethyl 2-[(ethoxycarbonothiolyl)thio]propanoate; CTA4—O-ethyl S-(1-phenylethyl)dithiocarbonate; CTA5—dibenzyl trithiocarbonate; CTA6—4-cyano-4-{[(ethylthio)carbonothioyl]thio})pentanoic acid.

We studied the controlled radical polymerization of a nonconjugated 1-vinyl-1,2,4-triazole monomer in the presence of AIBN as an initiator and chain transfer agent of the general formula Z–C(=S)–S–R, or Z–S–C(=S)–S–R differing in the structure of the stabilizing group Z and the leaving group R: dithiocarbamates—CTA1 and CTA2, xanthates—CTA3 and CTA4, and trithiocarbonates—CTA5 and CTA6 (Figure 1).

**Scheme 3:**
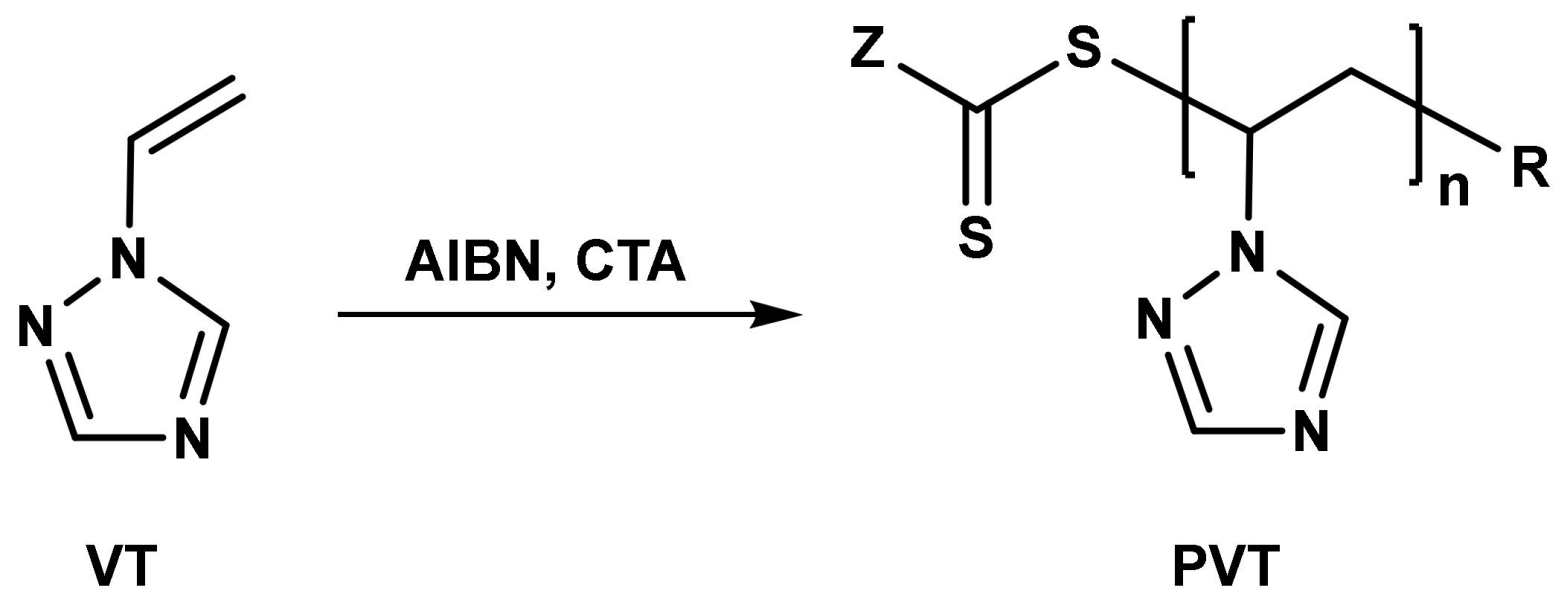
The synthesis of the poly(1-vinyl-1,2,4-triazole) controlled radical polymerization.

The controlled radical polymerization of VT was carried out in the presence of AIBN as the initiator and chain transfer agents CTA1–CTA6 in the ratio [AIBN]:[CTA] = 1:5 mol in methanol at 60 °C for 24 h (Scheme 3, Table 1).

**Figure 2:**
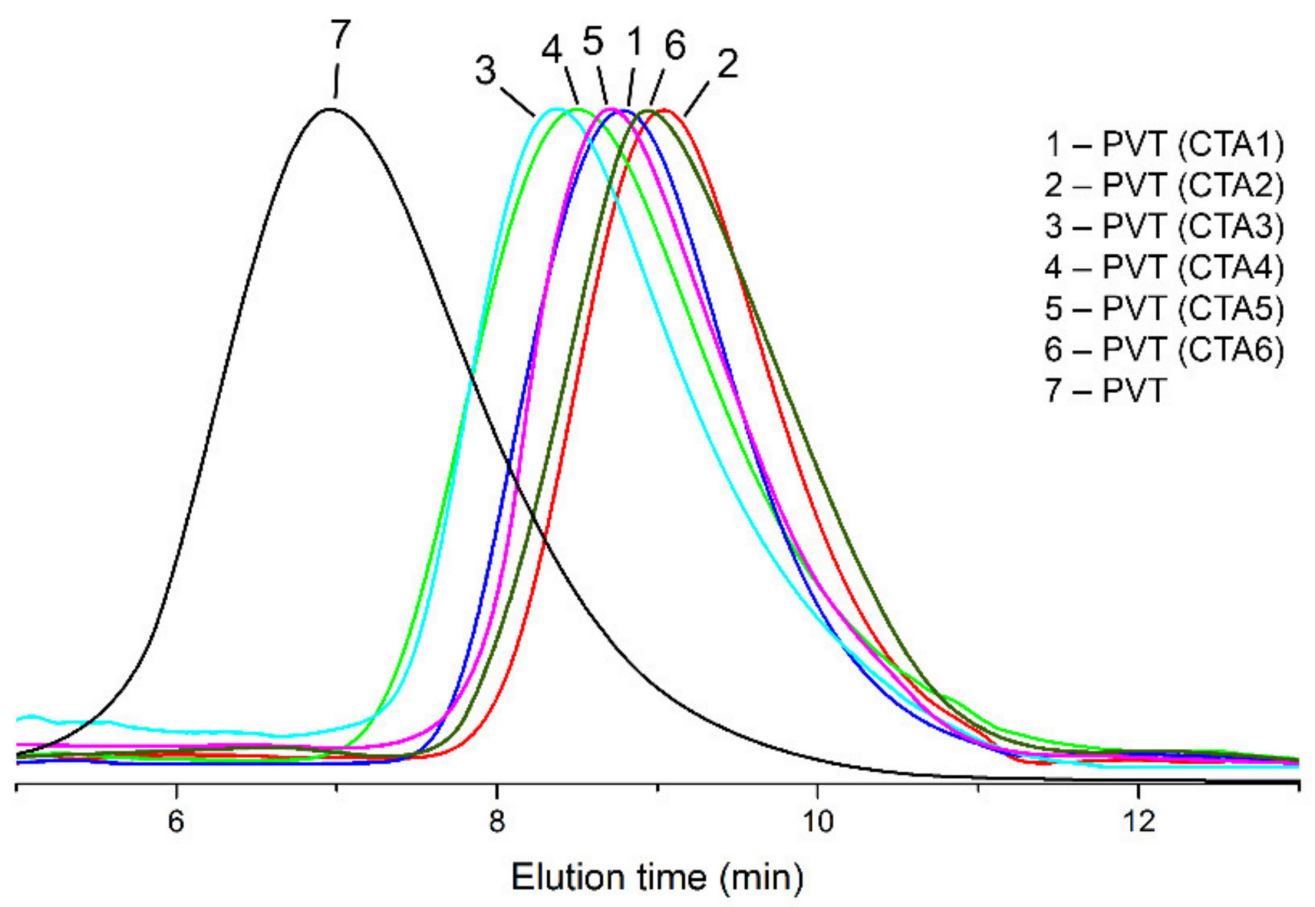
The GPC curves of PVT: 1–6—obtained by controlled radical polymerization in the presence of CTA1–CTA6; 7—obtained by free radical polymerization in methanol.

A comparative analysis of the effectiveness of CTAs of different natures in the controlled radical polymerization of VT under the same conditions was carried out (Figure 2). The data obtained indicate that all of the chain transfer agents used provided controlled radical polymerization and led to the production of narrowly dispersed polymers. The use of CTA3 and CTA4 (xanthates) resulted in PVT with the highest molecular weight and wide polydispersity. CTA5 and CTA6 (trithiocarbonates) showed average values of molecular weight and polydispersity among the studied agents. CTA1 and CTA2 (dithiocarbamates), which led to the most narrowly dispersed polymers, were the most efficient in the addition–elimination exchange reactions. In this regard, further study of the controlled polymerization of VT in order to control the molecular weight of the narrowly dispersed PVT was carried out using CTA1 and CTA2.

Carrying out the controlled radical polymerization of VT, in contrast to free radical polymerization, led to a decrease in the average molecular weights and the formation of narrowly dispersed polymers (Figure 2). The high reactivity of the growing 1-vinyl-1,2,4-triazole macro-radicals as well as the corresponding unfavorable transfer and polymer-chain termination reactions were suppressed by the RAFT process using active agents of the dithiocarbamate type.

The effect of the natures of the RAFT agent, solvent, molar ratio of the initiator AIBN and CTA on the monomer conversion, molecular weight, and polydispersity of the resulting poly(1-vinyl-1,2,4-triazole) were studied (Table 2).

The use of methanol and DMF was due to the good solubility of the PVT and RAFT agents in these solvents. The molar ratio of [AIBN]:[CTA] ranged from 1:1 to 1:10. Regardless of the CTA used and the polymerization conditions, the polymer conversion was more than 98%, and the average molecular weight of the PVT varied in the range from 11 to 61 kDa (Table 2). The use of methanol as a medium compared to DMF led to the production of polymers with a narrower polydispersity in the entire range of the [AIBN]:[CTA] ratios studied, which indicates a more efficient occurrence of the addition–elimination exchange reactions. With an increase in the ratio of AIBN to CTA from 1:1 to 1:10, the polydispersity of the resulting polymers decreased from 1.37–1.59 to 1.16–1.42, depending on the solvent and CTA. This indicates the inhibition of the chain growth reactions, leading to a decrease in the average molecular weights of the polymers and a more uniform increase in the degree of polymerization of the active macromolecular chains. The predominance of the chain transfer agent over the initiator increases the efficiency of control over the polymerization reaction by reducing the instantaneous concentration of radicals and chain termination reactions.

In order to control the molecular weight of PVT, studies were carried out at different ratios of [M]:[CTA] in the range from 100 to 400 at a constant ratio of [AIBN]:[CTA] = 1:2.5 (Table S1). According to GPC, the VT conversion was 98–99%. At the ratio [M]:[CTA] = 400:1, PVT was characterized by the highest molecular weights in the range of 47–59 kDa and the highest PDI of 1.21–1.28, depending on the solvent and CTA. As the ratio decreased to [M]:[CTA] = 100:1, the molecular weight and polydispersity decreased to 19–21 kDa and 1.16–1.21, respectively. Thus, the linear character of the dependence of the molecular weights on [M]:[CTA] indicates that the polymerization process is controlled.

The obtained results indicate that CTA2 is a more efficient RAFT agent in the controlled radical polymerization of VT compared to CTA1. CTA1 and CTA2 have the same leaving group (R = –CH_2_C≡N), so the efficiency of the reinitiation step for both chain transfer agents is close. The difference between CTA1 and CTA2 lies in the stabilizing group (Z), which ensures the stabilization of the macromolecular chain in the inactive (sleeping) state. Based on this, the difference in the molecular weight and dispersion of PVT is due to the influence of this fragment.

The phenyl fragment of CTA1 leads to the formation of a more stable polyCTA1, which participates less efficiently in the addition–fragmentation exchange reactions. This leads to an increase in the rates of macromolecular chain growth reactions.

The pyridine fragment of CTA2 increases the total electronegativity of the stabilizing group of polyCTA2. This probably leads to the destabilization of the (-C=S) bond and easier attachment of the growing macroradical to polyCTA2. As a result, polyCTA2 had higher activity and a higher transfer coefficient compared to polyCTA1, which led to a decrease in the growth reaction rates and a decrease in M_n_ and PDI.

**Figure 3:**
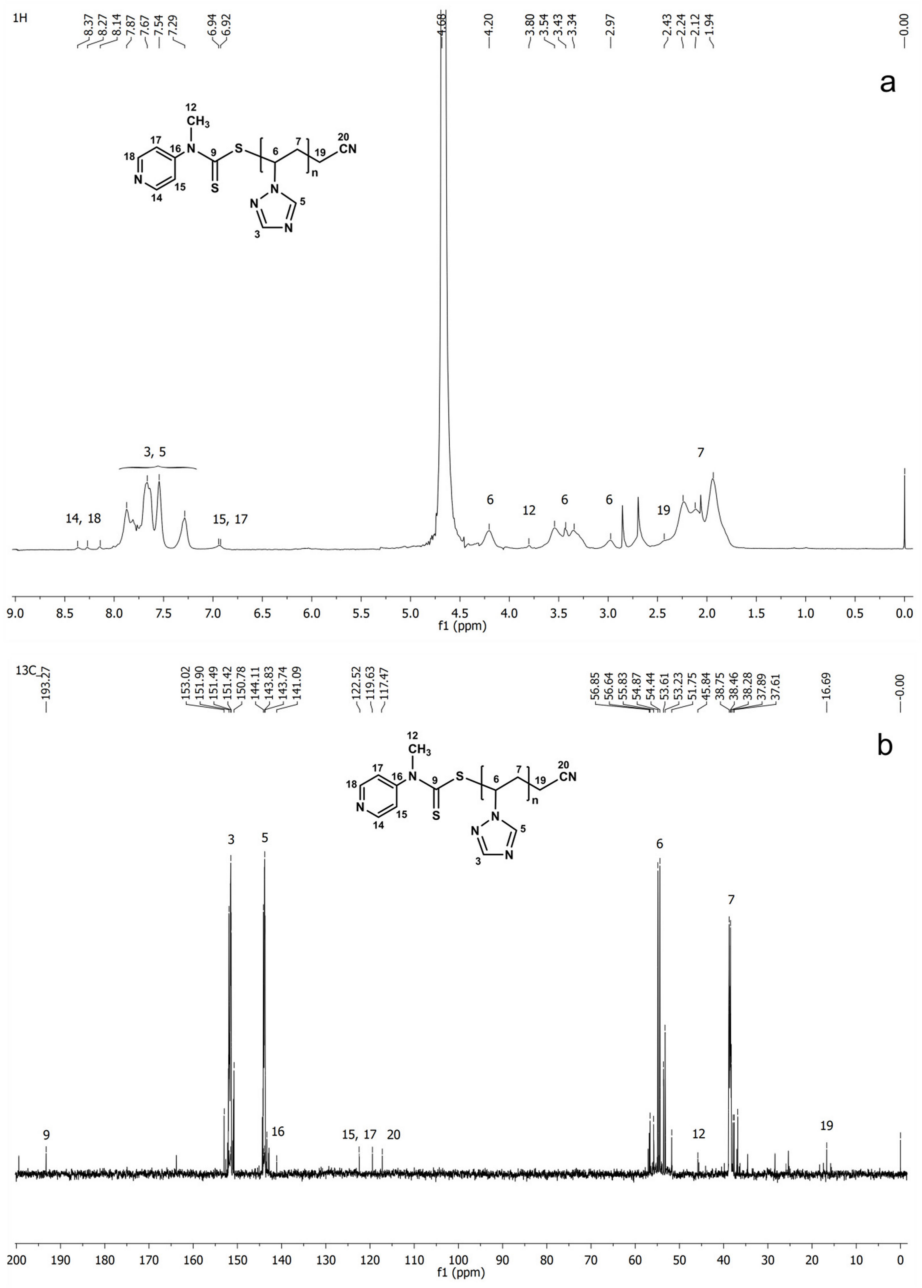
The ^1^H (**a**) and ^13^C (**b**) NMR spectra of PVT (Table 2, 12) in D_2_O.

The synthesized PVT was characterized by ^1^H and ^13^C NMR spectroscopy. The ^1^H NMR spectrum of the poly(1-vinyl-1,2,4-triazole) obtained at [AIBN]:[CTA2] = 1:10 in DMF is shown in Figure 3a. The signals characteristic of protons of the N-vinyl group of the monomer at 5.67 and 5.08 ppm completely disappeared in the ^1^H NMR spectrum of the homopolymer. New broadened signals showed up in the region of 4.20–3.34 and 2.34–1.94 ppm, which were assigned to the proton resonance in the methyne and methylene groups in the polymer backbone, respectively. The signals at 7.87–7.29 ppm belonged to the protons of the triazole ring. In addition, the ^1^H NMR spectrum contained signals at 8.37–8.14 and 6.94 ppm, which were related to the protons of the pyridine ring of CTA2. The methyl proton signals of the dithiocarbamate group of CTA2 were visible at 3.80 ppm. The signal attributable to the methylene protons of the NC–CH_2_ R-group was detected at 2.43 ppm. Such signals were not observed in the ^1^H NMR spectrum of the PVT synthesized by free radical polymerization (Figure S1a).

In the ^13^C NMR spectrum of the PVT, the signals of the triazole ring carbons were detected at 153.02–150.78 ppm and 144.11–141.09 ppm (Figure 3b). The chemical shifts at 128.87 and 105.22 ppm related to the vinyl carbons disappeared in the ^13^C NMR spectra of the synthesized PVT, whereas new signals for the polymer backbone with the methyne and methylene group carbons were observed in the region of 56.85–51.75 and 38.75–37.61 ppm, respectively. In addition to these peaks, a carbon atom signal was visible at 193.27 ppm, which corresponded to the carbon atom of the dithiocarbamate group of CTA2. Resonance signals at 117.47 and 45.84 ppm were related to the carbon atoms of the cyano and methyl groups of CTA2, respectively. There were no similar signals in the ^13^C NMR spectrum of the PVT synthesized by free radical polymerization (Figure S1b).

Thus, the NMR data confirm the successful preparation of poly(1-vinyl-1,2,4-triazole) and indicate that the polymer chain end is capped by CTA fragments as expected, according to the general mechanism of the RAFT process.

Kinetic studies of VT radical polymerization in the presence of [AIBN]:[CTA2] = 1:2 in DMF at 60 °C were carried out in order to confirm the controlled nature of the polymerization. The conversion and molecular weight characteristics of PVT from the time of polymerization were controlled. The conversion, average molecular weight, and polydispersity of the homopolymer in the reaction mixture were determined by GPC.

**Figure 4:**
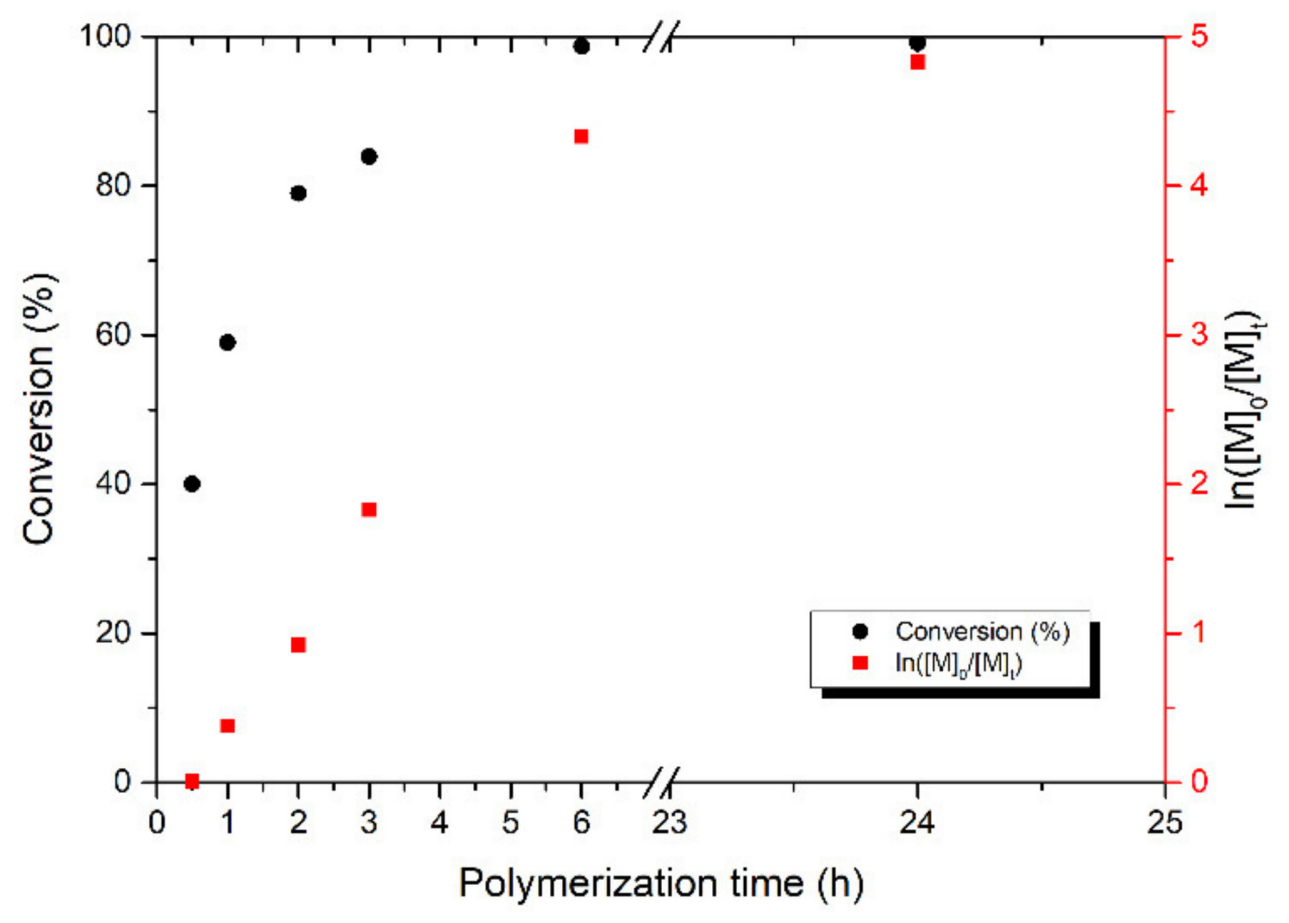
The time dependence of the VT conversion (circles) and first-order kinetics (squares) of 1-vinyl-1,2,4-triazole polymerization with [AIBN]:[CTA2] = 1:2 at 60 °C.

Monomer conversion and the ratio of the monomer concentration over time as a function of reaction time is shown in Figure 4. With an increase in the polymerization time, an increase in the monomer conversion is observed. The highest reaction rate was observed in the time interval from one to three hours. When it reached six hours, the reaction was almost complete. When the reaction was carried out for 24 h, the conversion reached 99%.

**Figure 5:**
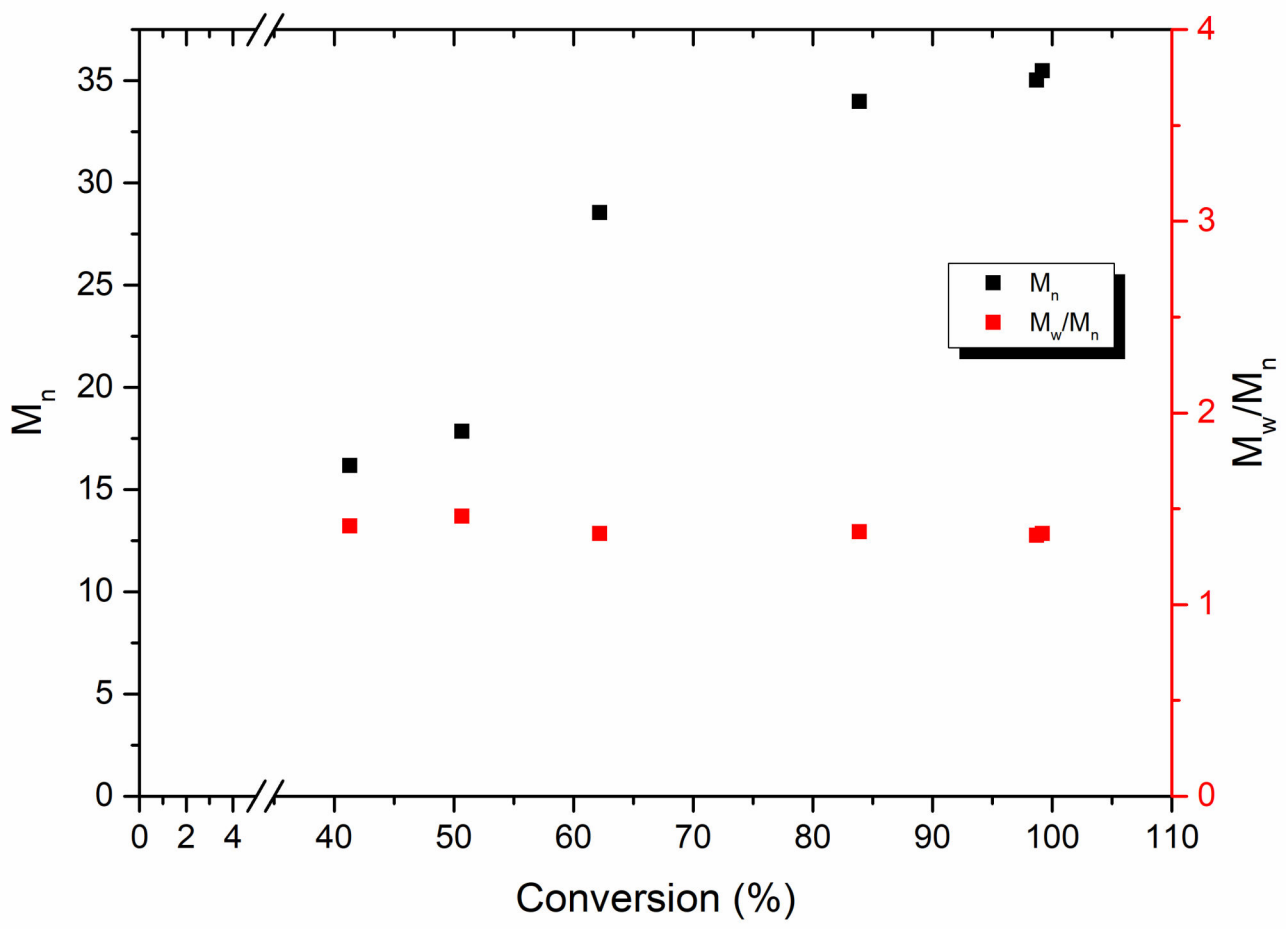
Dependence of the average molecular weight (M_n_) (black squares) and polydispersity M_w_/M_n_ (red squares) on the conversion during the polymerization of 1-vinyl-1,2,4-triazole, [AIBN]:[CTA2] = 1:2 mol at 60 °C.

The dependence of the average molecular weight and polydispersity on the monomer conversion is shown in Figure 5. The data showed that throughout the entire process of VT polymerization, the molecular weight of the polymer increased linearly with an increase in conversion, which indicates that the polymerization process proceeds in a controlled mode in the presence of CTA2.

**Figure 6:**
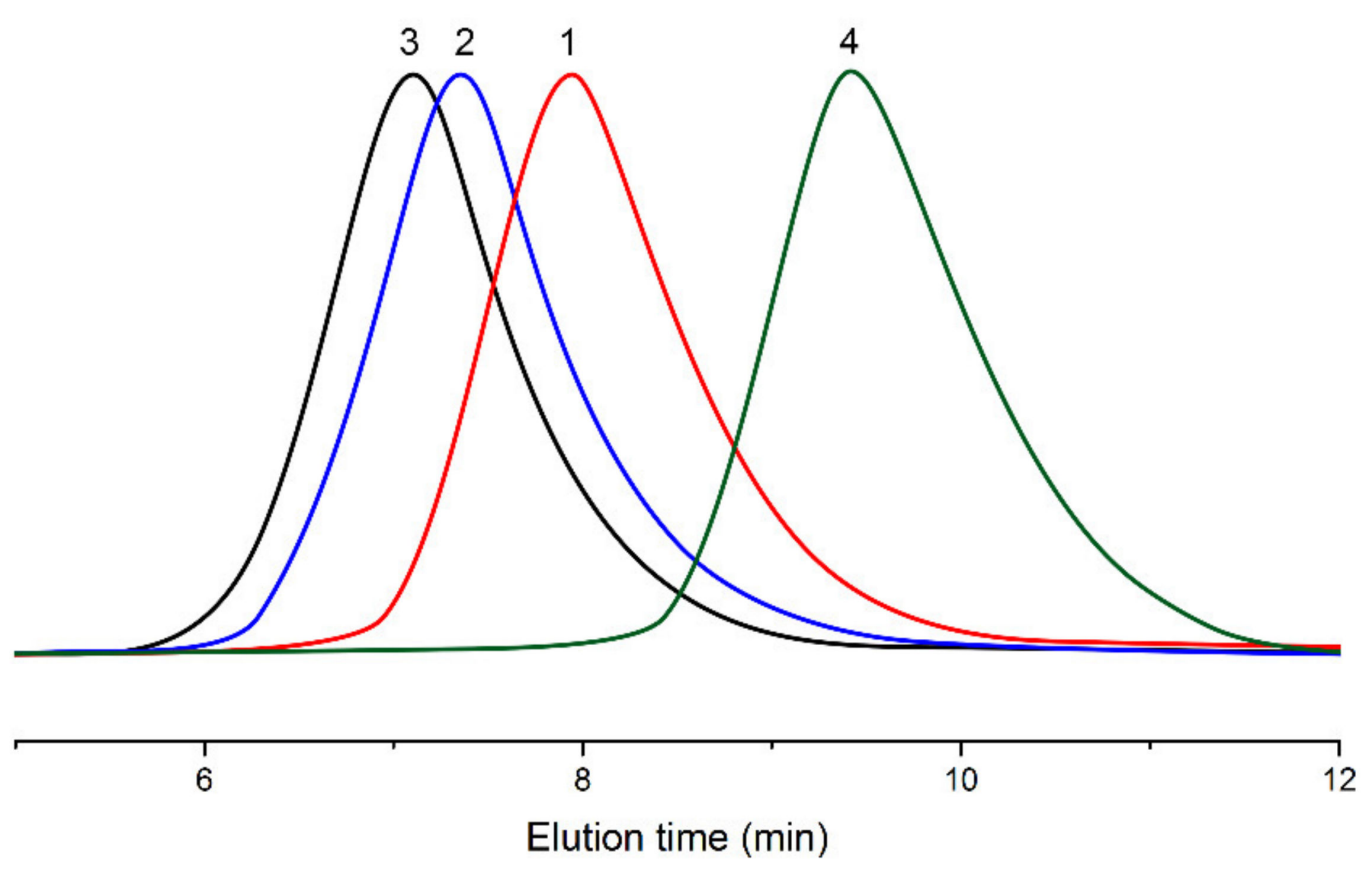
GPC curves: 1—polymer 1, Table S1 (Mn = 42,000, PDI = 1.23), 2—polymer 2, Table S1 (Mn = 61,000, PDI = 1.27), 3—polymer 3, Table S1 (Mn = 83,000, PDI = 1.13), 4—polymer 16, Table 2 (Mn = 11,000, PDI = 1.34).

An important criterion for proving the controlled nature of polymerization is the ability to further increase the polymer chain, which is in an inactive state. For this purpose, the resulting and isolated polymer PVT (Table 2, 16) was used as the macromolecular chain transfer agent polyCTA. AIBN was used as the polyCTA activator. The VT monomer was added to extend the macromolecular chain. The reaction was carried out for six hours. The resulting polymer PVT (Table S1, 17–19) was analyzed by GPC and compared with the original polymer PVT (Table 2, 16; Figure 6). The obtained data indicate that the molecular weight of the polymer increased from 11,000 to 42,000 Da ([M]:[polyCTA] = 400:1), to 61,000 Da ([M]:[polyCTA] = 600:1) and to 83,000 Da ([M]:[polyCTA] = 800:1) (Table S1).

## 4. Conclusions

The controlled radical polymerization of 1-vinyl-1,2,4-triazole in the presence of AIBN as an initiator and dithiocarbamates, xanthates, and trithiocarbonates as the chain transfer agents was studied. Dithiocarbamates—cyanomethyl methyl(phenyl)dithiocarbamate and cyanomethyl methyl(pyridine-4-yl)dithiocarbamate—proved to be the most effective chain transfer agents. The use of methanol as a medium compared to DMF led to polymers with a narrower polydispersity. The study of RAFT polymerization at various [M]:[CTA] ratios indicates a linear dependence of the molecular weight, which confirms the controlled mode of VT polymerization. Regardless of the polymerization conditions, the monomer conversion was greater than 98%. The average molecular weight of the synthesized PVT was in the range from 61,000 to 11,000 Da while the polydispersity reached 1.16. The structural analysis performed via the ^1^H and ^13^C NMR experiments revealed that the PVT chains contain CTA functional groups. The retention of activity of the PVT to chain transfer synthesized by the RAFT polymerization method was confirmed by successful chain extension.

## Figures and Tables

**Table 1:** The polymerization of 1-vinyl-1,2,4-triazole using CTA1–CTA6 in methanol at 60 °C for 24 h, [AIBN]:[CTA] = 1:5 *, [M]:[CTA] = 100:1.

Entry	CTA	Conversion, % **	M_n_, Da	PDI
1	CTA1	99	20,000	1.26
2	CTA2	>99	17,000	1.20
3	CTA3	97	25,000	1.38
4	CTA4	97	24,000	1.35
5	CTA5	95	21,000	1.32
6	CTA6	96	18,000	1.30

* AIBN 1 × 10^−2^ mol/L, [M] = 5.26 mol/L. ** Conversion determined by GPC.

**Table 2:** The synthesis conditions and characteristics of poly(1-vinyl-1,2,4-triazole) obtained by the RAFT polymerization in DMF or MeOH, at different ratios of AIBN and CTA at 60 °C for 24 h *.

Entry	CTA	Solvent	[AIBN]:[CTA]	Conversion, % **	M_n_, Da	PDI
1	CTA1	DMF	1:1	99	61,000	1.59
2			1:2.5	98	45,000	1.45
3			1:5	98	22,000	1.46
4			1:10	98	14,000	1.42
5		MeOH	1:1	>99	49,000	1.53
6			1:2.5	98	34,000	1.41
7			1:5	99	20,000	1.26
8			1:10	99	13,000	1.24
9	CTA2	DMF	1:1	>99	44,000	1.46
10			1:2.5	>99	26,000	1.37
11			1:5	>99	18,000	1.31
12			1:10	98	13,000	1.25
13		MeOH	1:1	99	27,000	1.37
14			1:2.5	>99	26,000	1.27
15			1:5	>99	17,000	1.20
16			1:10	>99	11,000	1.16

* [M] = 5.26 mol/L (in DMF and MeOH), [AIBN] 1 × 10^−2^ mol/L. **—Conversion determined by GPC.

## Data Availability

Not applicable.

## Supplementary Materials

The following supporting information can be downloaded at: https://www.mdpi.com/article/10.3390/polym14102029/s1, Table S1. Characteristics of poly(1-vinyl-1,2,4-triazole) obtained by chain addition to polyCTA at various ratios [M]:[polyCTA] in DMF at 60 °C for 6 h; Figure S1. ^1^H and ^13^C NMR spectra of poly(1-vinyl-1,2,4-triazole) synthesized by free radical polymerization; Table S2. Effect of [M]:[CTA] ratio on RAFT polymerization of VT at constant [CTA]:[AIBN] concentration ratio at 60 °C for 24 h in DMF and methanol.
