# Supplementary material for: Dithiocarbamates as Effective Reversible Addition–Fragmentation Chain Transfer Agents for Controlled Radical Polymerization of 1-Vinyl-1,2,4-triazole
Source: Polymers (Basel). 2022 May 16;14(10):2029. doi: 10.3390/polym14102029 (PMC9147191; doi:10.3390/polym14102029)
Supplement: Supplementary file 1 [file polymers-14-02029-s001.zip › polymers-1727651-supplementary.pdf]

Supplementary Materials

Dithiocarbamates as Effective Reversible Addition–Fragmentation Chain Transfer Agents for Controlled Radical Polymerization of 1-Vinyl-1,2,4-triazole

Alexander S. Pozdnyakov*, Nadezhda P. Kuznetsova, Tatyana A. Semenova, Yuliya I. Bolgova, Anastasia A. Ivanova, Olga M. Trofimova, and Artem I. Emel'yanov

A.E. Favorsky Irkutsk Institute of Chemistry, Siberian Branch of the Russian Academy of Sciences, 1 Favorsky Str., 664033 Irkutsk, Russia

* Corresponding author. E-mail address: pozdnyakov@irioch.irk.ru

Table S1. Characteristics of poly(1-vinyl-1,2,4-triazole) obtained by chain addition to polyCTA at various ratios [M]:[polyCTA] in DMF at 60 °C for 6 h.*

Entry	[M]:[polyCTA]	Conversion, %	$M_{n, theory}$, Da	M_n, Da	PDI
1	400	99	38000	42000	1.23
2	600	99	57000	61000	1.27
3	800	98	75000	83000	1.34

* [AIBN]:[CTA] = 1:2.5, [M] = 4.97 mol/L, polyCTA – Table 2, 16. Number-average molecular weight (M_n) and molecular mass distribution (M_w/M_n) were measured by GPC. The theoretical molecular weight ($M_{n, theory}$)=(MW of VT)×[M]:[polyCTA]×conv.+(MW of polyCTA). Number-average molecular weight (M_n) and molecular weight distribution (M_w/M_n) were measured by GPC.

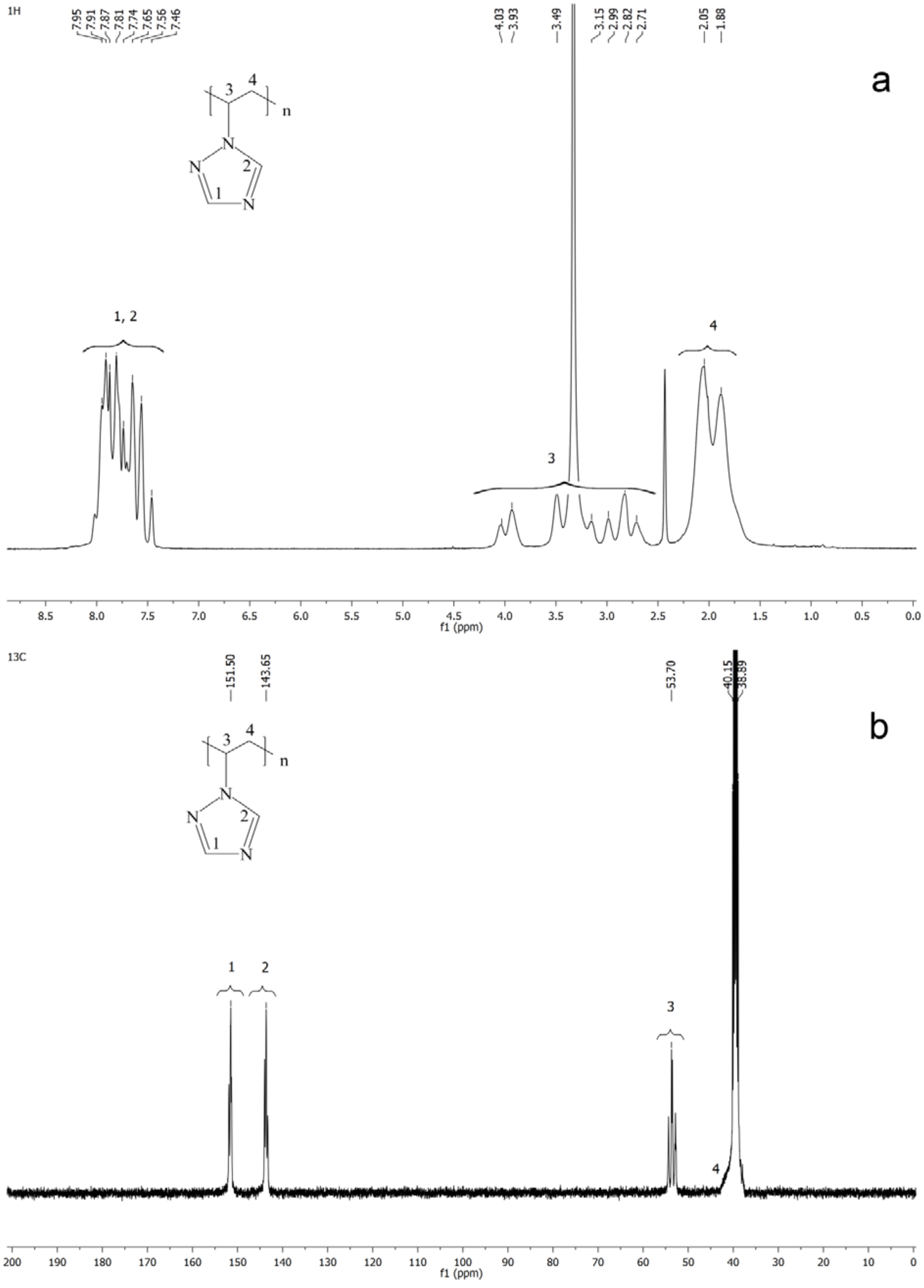

Figure S1. ^{1}H (**a**) and ^{13}C (**b**) NMR spectra of poly(1-vinyl-1,2,4-triazole) synthesized by free radical polymerization.

Table S2. Effect of [M]:[CTA] ratio on RAFT polymerization of VT at constant [CTA]:[AIBN] concentration ratio at 60 °C for 24 h in DMF and methanol.*

Entry	CTA	Solvent	[M]:[CTA]	Conversion, %	$M_{n, theory}$, Da	M_n, Da	PDI
1	CTA1	DMF	100	98	9300	21000	1.21
2			200	98	19000	37000	1.25
3			300	98	28000	49000	1.27
4			400	98	37000	51000	1.28
5		MeOH	100	>99	9500	20000	1.19
6			200	99	19000	35000	1.21
7			300	99	28000	42000	1.22
8			400	99	37000	47000	1.24
9	CTA2	DMF	100	>99	9500	20000	1.17
10			200	>99	19000	35000	1.19
11			300	>99	28000	47000	1.21
12			400	98	37000	59000	1.25
13		MeOH	100	98	9400	19000	1.16
14			200	>99	19000	32000	1.18
15			300	>99	28000	44000	1.19
16			400	>99	38000	55000	1.21

* [AIBN]:[CTA] = 1:2.5, [M] = 4.97 mol/L. Number-average molecular weight (M_n) and molecular mass distribution (M_w/M_n) were measured by GPC. The theoretical molecular weight ($M_{n, theory}$)=(MW of VT)×[M]:[CTA]×conv.+(MW of CTA). Number-average molecular weight (M_n) and molecular weight distribution (M_w/M_n) were measured by GPC.
